# Supplementary material for: Assessment of Protein Content and Phosphorylation Level in Synechocystis sp. PCC 6803 under Various Growth Conditions Using Quantitative Phosphoproteomic Analysis
Source: Molecules. 2020 Aug 6;25(16):3582. doi: 10.3390/molecules25163582 (PMC7466104; doi:10.3390/molecules25163582)
Supplement: Supplementary file 1 [file molecules-25-03582-s001.zip › Supplementary_Materials_rev/Supplementary_Materials_Figures.pdf]

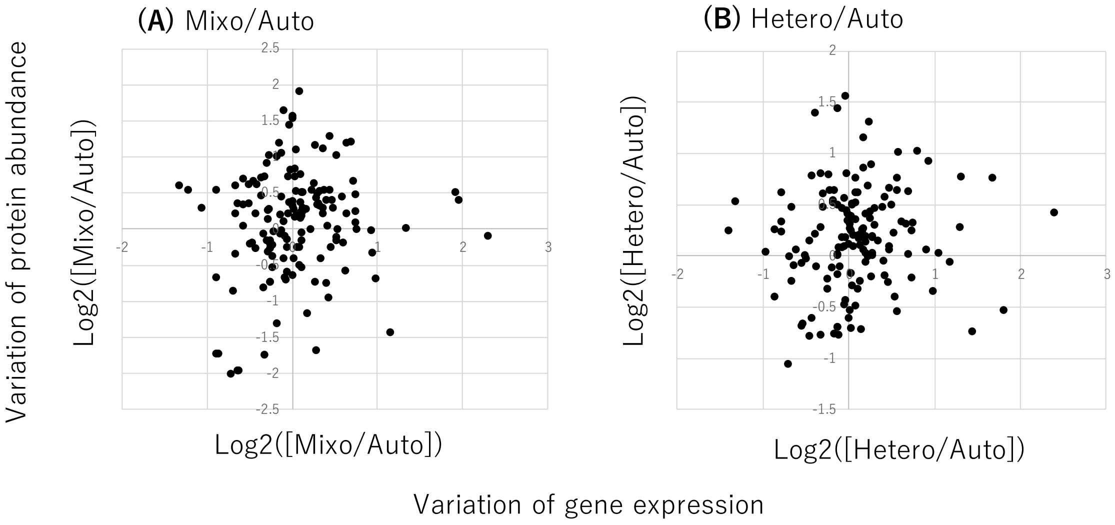

Figure S1. Comparison between variations in gene expression and protein abundance. Gene expression dataset was obtained from literature [18]. Protein abundance dataset was obtained from this study using the identical strain and the culture conditions.

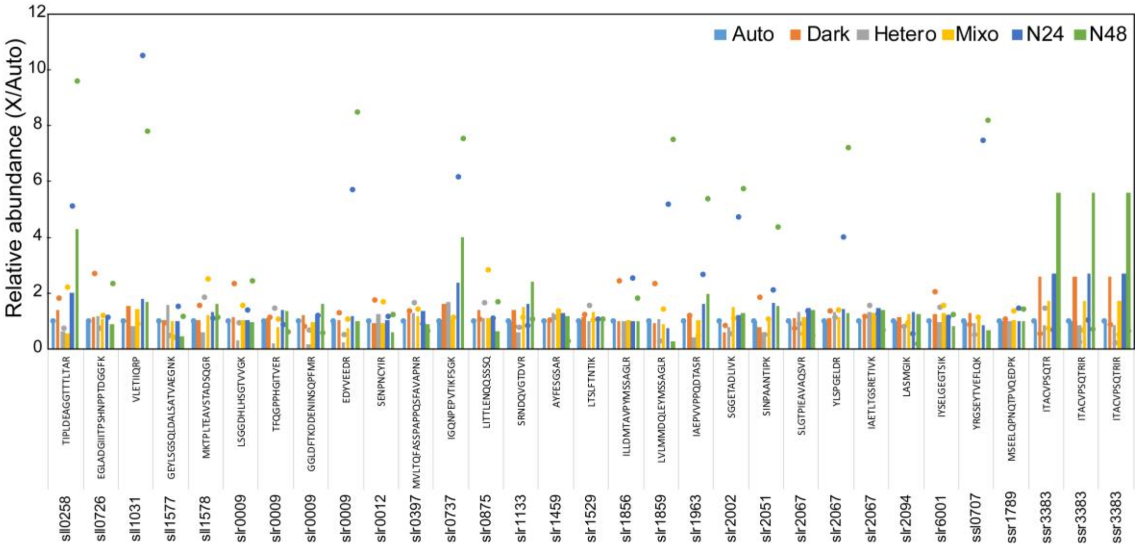

Figure S2. Relative abundance of protein and phosphoprotein. Bars show the protein abundance. Dots show the phosphopeptide abundance. Each value represents the mean of three independent experiments.
